# Subjective Cognitive Dysfunction in Chronic Illness: A Systematic Review and Meta-Synthesis

**DOI:** 10.1177/01939459241272039

**Published:** 2024-08-19

**Authors:** Heather Cuevas, Elizabeth Heitkemper, Jeeyeon Kim

**Affiliations:** 1 School of Nursing, The University of Texas at Austin, Austin, TX, USA

**Keywords:** cognitive impairment, cognitive dysfunction, health behavior/symptom focus, meta-synthesis, qualitative methods

## Abstract

**Background:**

Qualitative studies have examined the experiences of subjective cognitive dysfunction in specific populations or specific disease stages, but there has not yet been a systematic synthesis and evaluation of findings related to perceptions of subjective cognitive dysfunction in nondementia-related chronic illnesses.

**Objective:**

The aim of this study was 2-fold: (1) to undertake a systematic review of experiences of subjective cognitive dysfunction in people with nondementia-related chronic disease and (2) to develop an explanatory framework to describe the experiences of living with subjective cognitive dysfunction.

**Methods:**

Four databases were systematically searched for studies on subjective cognitive dysfunction up to June 2023. Qualitative synthesis was conducted on the final sample (N = 25) using Sandelowski’s adaptation of Nobilt and Hare’s reciprocal transactional analysis method. Critical appraisal was completed using the Critical Appraisal Skills Programme checklist.

**Results:**

Through constant comparison of key concepts, findings were organized within 4 interrelated themes that informed a conceptual explanatory model of adapting to living with subjective cognitive dysfunction: (1) symptoms, (2) health care, (3) perceptions of self, and (4) relationships. Participants highlighted how subjective cognitive dysfunction affected interactions in health care settings and involved other symptoms that in turn complicated meaning, self-enhancement, and mastery.

**Conclusions:**

Our model of the process of adapting provides a new way to conceptualize cognitive dysfunction in chronic illness and suggests opportunities for health care professionals to support patients and their families. The results highlight the need for more research to better understand the role of subjective cognitive dysfunction in nondementia-related chronic illnesses.

The review protocol was registered in PROSPERO (CRD42021231410).

Current standard assessments of cognitive dysfunction do not include subjective (eg, self-reported) data. Instead, cognitive dysfunctions, defined as decreased cognitive function in one or more domains such as memory or attention, are assessed with standard neuropsychological tests that consist primarily of performing simple tests to demonstrate mental function.^1^ However, by not including people’s self-reported experiences of cognitive function and the perceived effects on daily life in these assessments, there is a clear gap in understanding cognitive changes and their impacts on individuals’ health and quality of life. Furthermore, subjective cognitive dysfunction has been shown to be a risk factor for dementia, sometimes presenting before objective impairments especially in those with chronic illnesses—even in nondementia-related chronic conditions not typically associated with cognitive dysfunction such as diabetes, chronic obstructive pulmonary disease, and hypertension.^2^ For example, 27.1% of adults aged 45 to 65 years who have coronary artery disease report subjective cognitive dysfunction, whereas in healthy adults aged 65 years and older, the prevalence is 18.7%.^3^ It has been shown that subjective cognitive dysfunction can affect daily self-management of chronic conditions,^4,5^ as well as individuals’ quality of life.^6^ Thus, it is critical to synthesize how subjective changes affect individuals.

## Subjective Experiences of Cognitive Dysfunction

Examining individuals’ own perspectives on symptoms such as forgetfulness or confusion and on the impacts and trajectories of their cognitive dysfunction is needed. For these reasons, there has been an increase in both qualitative and quantitative research on subjective cognitive dysfunction in persons at risk for neurodegenerative disorders including dementia. In one systematic review of the quantitative literature,^7^ almost every study was found to have used a unique approach to assess subjective cognitive function (32 out of 37 studies). This heterogeneity of instrumentation inhibits the sharing of data and the generalizability of quantitative results. The use of common data elements for subjective cognitive function in persons with nondementia-related chronic illness could accelerate the development and testing of interventions. Studies reviewed by Hill et al^7^ used 28 measures to assess subjective cognitive function with little overlap among them. In a prior review and meta-analysis of studies (n = 53; 20 319 participants), Crumley et al^8^ examined the association between objective and subjective cognitive function in normatively aging adults without chronic illnesses and found that subjective cognitive function accounted for less than 1% of the performance in objective measures. However, Crumley et al^8^ included studies that used 5 specific measures for subjective memory and likely excluded a large number of other studies, because there are no “gold-standard” assessments of subjective cognitive function.^7^ It has also been posited that affective symptoms like anxiety are positively related to subjective cognitive dysfunction and may be difficult to disentangle.^9^ However, a review of 58 studies^10^ examined the relationships between subjective cognitive function and affective symptoms and found that while they were strongly correlated, but little was known regarding the temporality of the relationships (ie, does subjective cognitive dysfunction precede anxiety and depression?). An additional review of 32 studies analyzing subjective cognitive dysfunction in nondementia-related chronic illness confirms this finding and found a strong association between subjective cognitive dysfunction and other patient-reported outcomes.^11^ The authors of both reviews concluded that more qualitative investigation into the experiences of subjective cognitive dysfunction was needed.

Qualitative studies have examined the experiences of subjective cognitive dysfunction in specific populations or specific disease stages,^4,5,12-41^ but there has not yet been a systematic synthesis and evaluation of findings related to perceptions of subjective cognitive dysfunction in nondementia-related chronic illnesses. Qualitative synthesis can be used to better understand health-related phenomena such as subjective cognitive dysfunction from the perspectives of the people who may at some point be targeted by interventions to improve those phenomena.^42^ If we recognize these individuals’ perspectives, it may be possible to achieve more effective behavior change.^43^

## Purpose

Therefore, the aim of the present study was 2-fold: (1) to undertake the first systematic review of experiences of subjective cognitive dysfunction in people with nondementia-related chronic disease; and (2) in a meta-synthesis of the findings of this review, to form an explanatory framework that could describe the experience of living with subjective cognitive dysfunction.

## Methods

In this review, we use the method of qualitative synthesis adapted by Sandelowski et al^44^ from Noblit and Hare’s^45^ reciprocal translational analysis, to integrate separate studies into a larger “whole.” The theoretical basis for this study is Turner’s theory of social explanation,^46^ which posits that interpretation of phenomena progresses through comparison. The goal is to examine differences and similarities of individual studies to develop a theory that goes beyond immediate research contexts. With this in mind during the thematic analysis of qualitative data, we inductively identified themes extracted across studies, and we identified similarities and offered novel interpretations not found in any one study alone. This meta-synthesis follows the guidelines for enhancing transparency in reporting the synthesis of qualitative research (ENTREQ).^47^ The review protocol is registered in PROSPERO (CRD42021231410).

### Search

To search the literature for studies on experiences of subjective cognitive dysfunction in people with a nondementia-related chronic condition, we adopted a comprehensive strategy with the assistance of a health-sciences librarian. Using the guidelines for systematic reviews by Petticrew and Roberts,^48^ we initially selected broad terms for the population of interest (eg, “chronic illness,” “non-dementia”), types of studies (eg, “qualitative,” “mixed methods”), and the phenomenon of interest (“subjective cognitive dysfunction”). From Medline and CINAHL we obtained words and terms, including index terms, to describe our concepts. A list of keywords was thus developed to inform our full search of the CINAHL, Medline, EMBASE, and PubMed databases.

### Inclusion/Exclusion Criteria

**Figure 1.**
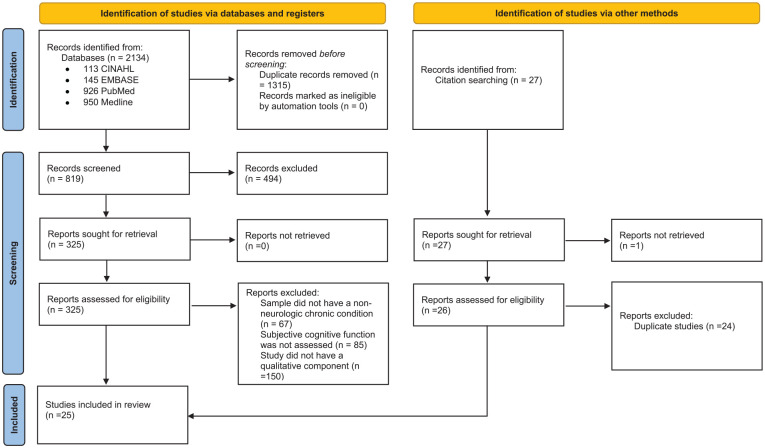
Preferred Reporting Items for Sytematic reviews and Meta-analyses (PRISMA) flow diagram.

Inclusion criteria for the studies were as follows: (1) qualitative studies or mixed-methods studies that reported primary qualitative data in their results; (2) studies that explicitly addressed the experiences of subjective cognitive dysfunction; (3) studies with participants who had chronic nondementia-related illnesses, without diagnoses of dementia or mild cognitive impairment; (4) studies published in English; (5) studies published in peer-reviewed journals; and (6) studies published through June 2023. Excluded studies were (1) quantitative studies; (2) conference abstracts; (3) review articles; (4) studies exclusively of participants with diagnoses of dementia or mild cognitive impairment; (5) studies that addressed cognitive function only with objective neuropsychological tests. (See Supplemental Material A for details.). Duplicates were removed after the initial search, and 2 investigators independently screened the literature for eligible articles. First, abstracts and titles were screened for inclusion, and those considered relevant were advanced to full-text review. Full-text studies were then reviewed by 2 investigators, and disagreements were resolved until 100% agreement was reached by the 3 authors (see Figure 1).^12^ Reference lists in the included articles were also scanned for any additional pertinent literature.

### Quality Appraisal

The included studies were independently reviewed by the authors using the Critical Appraisal Skills Programme (CASP) Checklist.^13^ This 10-item tool rates each domain of quality as “yes,” “no,” or “can’t tell.” The domains comprise aims, methodology, design, sampling, data collection, reflexivity, ethics, data analysis, findings, and contribution (see Table 1).

**Table 1.** Study Characteristics.

Study	Aim	Participants	Data Collection	Analysis	Theme(s)
First author Year	Study aim(s)	Sampling approach Age range N by gender Race/ethnicity Chronic condition	Type Duration	Theory or framework Analysis method	Main themes/results
Alenljung 2019	To explore how women with manage everyday lives after mild stroke and cognitive problems	■ Purposive ■ 38-64 years ■ F = 10, M = 0 ■ NR ■ Cardiovascular disease	■ One-on-one semi-structured interviews ■ 35-60 minutes	■ None ■ Content analysis	■ Participants had to plan daily activities in new ways: the everyday was affected by symptoms. ■ Living strategies. ■ Social environment effects and changes.
Bolt 2020	To explore the lived experience of cognitive changes	■ Purposive via cancer support groups ■ 43-67 years ■ F = 13, M = 0 ■ NR ■ Head and neck cancer	■ One-on-one semi-structured interviews ■ 60-120 minutes	■ Braun andac Clarke’s framework for thematic analysis ■ Thematic analysis	■ Participants were unprepared for cognitive problems: “I would have told you about being forgetful, but I forgot; “It’s such a journey that much of it is uncharted.”
Callan 2022	To explore the lived experience of “brain fog” that can follow COVID-19.	■ Purposive from online support groups ■ 29-74 years ■ F = 42, M = 8 ■ White British = 30 ■ White other = 6 ■ Black = 2 ■ Asian = 5 ■ Nonresponse = 7 ■ Long COVID	■ Focus groups ■ 60-90 minutes	■ Neuroscience symptom burden and May’s burden of illness theory ■ Thematic analysis	■ Experiences of neurocognitive symptoms accounted for how the illness fluctuated and progressed over time. ■ Participants searched for physical mechanisms to explain their symptoms.
Chasco 2022	To better understand the lived experiences of cognitive complaints in patients with post-acute sequalae of SARS-CoV-2	■ Purposive from a clinic registry ■ 40-68 years ■ F = 10, M = 5 ■ NHW = 13, H = 2 ■ Long COVID	■ One-on-one semi-structured interviews ■ 26-83 minutes	■ None ■ Thematic analysis	■ “Brain fog” affected all subthemes (lack of support, social isolation, guilt, poor self-perception, fear of loss of income, and social stigma).
Crouch 2017	To explore how breast cancer survivors cope and self-manage cognitive changes	■ Purposive ■ 36-64 years ■ F = 13, M = 0 ■ NR ■ Breast cancer	■ One-on-one semi-structured telephone interviews ■ Average 22 minutes	■ None ■ Content analysis	■ Coping and self-management strategies were used. ■ Nonpharmacologic interventions were preferred.
Crowe 2020	To explore experiences of cognitive impairment	■ Purposive ■ 21-55 years ■ F = 10, M = 10 ■ NHW = 15, ■ AAPI = 1, ■ Maori = 1, ■ Other = 3 ■ Depression	■ One-on-one semi-structured interviews ■ NR	■ Diathesis stress model ■ Thematic analysis	■ Three main types of experiences of cognitive impairment were described: 1. Being stuck 2. Being preoccupied with thoughts 3. Being prevented from living to one’s potential
Cuevas 2017	To explore diabetes related cognitive problems	■ Purposive ■ 44-70 years ■ F = 5, M = 5 ■ NHW = 4, H = 4, ■ B = 2 ■ Type 2 diabetes	■ One-on-one semi-structured interviews ■ 35-80 minutes	■ None ■ Content analysis	■ Cognitive problems affected self-management. ■ Participants searched for advice on dealing with symptoms and maintenance of cognitive health.
Cuevas 2018	To describe the experiences of participants completing a cognitive rehabilitation intervention	■ Convenience ■ 40-70 years ■ F = 11, M = 8 ■ NHW = 6, B = 3, ■ H = 10 ■ Type 2 diabetes	■ Focus groups ■ 45 minutes	■ None ■ Content analysis	■ Before the intervention, participants did not tie their experiences of cognitive problems to diabetes. ■ After the intervention, the participants had expectations of cognitive change, used cognitive strategies, and described their effect on self-management.
Disler 2020	To explore views on cognitive impairment and screening	■ Convenience ■ 63-90 years ■ F = 6, M = 9 ■ NR ■ COPD	■ Focus groups ■ 35-65 minutes	■ None ■ Thematic analysis	■ Participants had limited awareness of the connection between cognitive change and COPD. ■ Cognitive change was seen as a normal part of aging. ■ Current strategies for self-management and cognitive function were described. ■ Most participants were not worried about cognitive testing.
Klymko 2011	To explore self-care experiences in African Americans with some cognitive difficulties	■ Purposive ■ 60-89 years ■ F = 6, M = 4 ■ B = 10 ■ Hypertension	■ One-on-one semi-structured interviews ■ ~60 minutes	■ None ■ Inductive content analysis	■ Self-care was found to be cognitively challenging. ■ Self-care challenges were consistent with the finding that 60% of the participants had difficulty with a cognitive task requiring complex cognitive skills.
Kudlicka 2018	To investigate how cognitive difficulties affect everyday life	■ Convenience ■ 65-77 years ■ F = 4, M = 7 ■ NR ■ Parkinson’s disease	■ One-on-one semi-structured interviews ■ NR	■ None ■ Inductive content analysis	■ Cognitive difficulties had a far-reaching impact on everyday life. ■ Significance depended on personal circumstances, such as the level of responsibilities of the person with Parkinson’s disease and the extent of available support.
Lelorian 2012	To explore cognitive processing in long-term breast cancer survivors	■ Random sample from a larger study ■ <60 years = 13, ■ 60-70 years = 13, ■ >70 years = 2 ■ F = 28, M = 0 ■ NR ■ Breast cancer	■ Face-to-face or telephone interviews ■ 18-178 minutes	■ None ■ Alceste analysis	■ One thematic class of post-traumatic growth was specific to women with high coping and social support and active cognitive processing. ■ Posttraumatic growth appeared most often as a conclusion of interviews rather than in response to the question about changes after cancer.
Mc Auliffe 2019	To explore the needs of people with multiple sclerosis who have self-reported cognitive deficits	■ Purposive ■ 23-59 years ■ F = 5, M = 2 ■ NR ■ Multiple sclerosis	■ Semi-structure telephone interviews ■ Average 30 minutes	■ None ■ Inductive thematic analysis	■ Three themes: 1. *Neglected symptoms*: participants’ frustrations about the importance given to cognition by health care providers. 2. *Impact on participation in daily occupations*: the everyday impacts of cognitive difficulties. 3. *Adaptations and adjustments to continued participation*: how participants manage, despite their difficulties.
Miebach 2018	To explore the occurrence of cognitive complaints in a clinical setting and compare those with depression and nondemented patients in a memory clinic	■ Purposive ■ 55-86 years ■ F = 26, M = 16 ■ NR ■ Depression	■ One-on-one semi-structured interviews ■ NR	■ None ■ Qualitative expert rating (theme absent vs present) and compared between the groups	■ Themes for complaints in patients with major depressive disorder align with depressive symptoms. ■ These themes appear to be different in part from the “cognitive complaint profile” of memory clinic patients.
Miebach 2019	To identify features of cognitive complaints in people with depression and healthy controls	■ Purposive ■ Mean 70.55 years ■ F = 10, M = 1 ■ NR ■ Depression	■ One-on-one semi-structured interviews ■ 13-20 minutes	■ None ■ Interpretive phenomenological analysis	■ Participants with major depression complained frequently about memory difficulties in comparison with healthy controls. ■ Many complaints seemed to emerge from depressive symptoms (ie, complaints were more related to a lack of drive and a psychical state of exhaustion), rather than from cognitive decline.
Munir 2011	To explore what support is available to help women understand the effects of chemotherapy	■ Purposive ■ 34-62 years ■ F = 31, M = 0 ■ NR ■ Breast cancer	■ One-on- one semi-structured interviews ■ NR	■ None ■ Content analysis	■ Problems with remembering tasks at work were most common. ■ Participants requested more information and support on cognitive difficulties. ■ From findings, potential interventions were identified: 1. Information and activities on cognitive strategies 2. Help with emotional distress associated with cognitive difficulties 3. Advice for families and employers.
Myers 2012	To describe and identify information women would find useful prior to chemotherapy and the onset of cognitive changes	■ Purposive ■ 25-65 years ■ F = 18, M = 0 ■ NR ■ Breast cancer	■ Focus groups and semi-structured interviews ■ Mean 60 minutes	■ None ■ Content analysis	■ “Life with chemobrain” was identified as the overarching theme in the study. ■ Three subthemes 1. *How I changed*: descriptions of participants’ experiences. 2. *How I cope*: coping strategies that participants employed. 3. *How to teach me*: methods and education participants would like to receive.
Pappadis 2019	To explore the perceptions of chronic poststroke cognition	■ Convenience ■ Mean 65.1 years ■ F = 12, M = 30 ■ NHW = 23, B = 11, H = 5, AAPI = 1 ■ Stroke—cardiovascular disease	■ Semi-structured interviews ■ 30-45 minutes	■ None ■ Thematic content analysis	■ The majority of participants (93%) reported cognition-related themes: 1. language and communication 2. memory 3. thinking abilities 4. comprehension 5. visuospatial processing 6. cognitive assessments and training 7. Nearly half of the participants reported an unmet need for cognitive or mood-related treatment.
Potrata 2010	To obtain an in-depth understanding of cognitive impairments and concerns described by patients with multiple myeloma	■ Purposive ■ 42-75 years ■ F = 5, M = 10 ■ NHW (British) = 11, ■ Asian (British) = 3 ■ Multiple myeloma	■ Semi-structured interviews ■ NR	■ None ■ Content analysis	■ Various cognitive impairments were observed and/or expressed in at least 10 of 15 patients. ■ In some patients, cognitive impairments significantly interfered with their personal and professional lives. ■ Patients used various coping strategies, from denial to using systems for counting medications, to cope with the results of their cognitive impairment.
Rust 2013	To examine cognitive changes in African American breast cancer survivors	■ Purposive ■ 46-60 years ■ F = 24, M = 0 ■ B = 24 ■ Breast cancer	■ Focus groups ■ 60 minutes	■ None ■ Grounded theory	■ Four themes were identified: 1. Concept of chemobrain 2. Variability of chemobrain among individuals 3. Stigma of chemobrain 4. Methods of coping 5. In addition, findings revealed that health professionals were not used as a resource to address the issues of chemobrain.
Sawyer 2016	To evaluate neurocognition after surviving sudden cardiac arrest	■ Convenience ■ 18-81 years ■ N = 157 ■ NR ■ Cardiac arrest—cardiovascular disease	■ Internet survey with open-ended questions ■ NR	■ None ■ Inductive thematic analysis	■ Forty-four percent of survivors indicated problems with memory after hospital discharge; there was a lack of information on memory issues at discharge.
Vaartio-Rajalin 2015	To identify factors affecting cognitive resources and knowledge expectations	■ Purposive ■ 18-87 years ■ N = 53 ■ NR ■ Cancer	■ Focus groups and semi-structured interviews ■ 56-87 minutes	■ None ■ Inductive content analysis	■ Critical moments for patients related to: 1. Diagnosis phase 2. Preparation of medical treatment plan 3. Living through the treatments 4. Getting rehabilitation as an outpatient 5. Facing the follow-up phase
Wu 2013	To describe the patients’ experiences of cognitive changes since starting androgen deprivation therapy	■ Convenience ■ 42-69 years ■ F = 0, M = 11 ■ NHW = 9, B 1, ■ Arab = 1 ■ Prostate cancer	■ Semi-structured telephone interviews ■ NR	■ None ■ Content analysis	■ Eight of the 11 participants reported impairments in the domains of concentration, information processing, verbal fluency, visual information processing/visuospatial function, memory, and executive dysfunction. ■ Neurobehavioral problems including neurofatigue and apathy were also reported.
Zheng 2017	To explore perceived cognitive impairment and relevant supportive care needs	■ Convenience ■ 18-60 years ■ F = 31, M = 0 ■ Chinese = 31 ■ Cervical cancer	■ Semi-structured interviews ■ 30-45 minutes	■ Myer’s model of chemotherapy related cognitive changes ■ Content analysis	■ Twenty women (64.5%) reported cognitive complaints. ■ The most common complaint was loss of concentration (n = 17, 85.0%). ■ Perceived contributing factors to these cognitive complaints included chemotherapy (n = 15, 75.0%), and ageing (n = 8, 40.0%). ■ These cognitive problems most commonly affected daily living (n = 20, 100%). ■ Common supportive care needs included symptom management strategies (n = 11, 55.0%) and counseling services (n = 8, 40.0%).

Abbreviations: AAPI: Asian or Pacific Islander; B: Black; COPD: chronic obstructive pulmonary disease; F: female; H: Hispanic; M: male; NHW: non-Hispanic white; NR: not reported.

### Data Extraction and Synthesis

Sandelowski et al’s^44^ guidelines for data extraction and synthesis of studies in health care enable one to interpret findings rather than simply compare them. With this method, comparison involves determining whether the same concept is found in multiple studies, whereas interpretation involves the construction of a larger picture—“lines of argument synthesis” from multiple disciplines. Texts are first coded line by line, and key concepts in each study are identified as constructs of the first order (participants’ quotations), second order (authors’ interpretations), and third order (synthesized from first and second order). Similarities and variances between the codes (first and second order constructs) are grouped into a hierarchy of descriptive themes, which are grouped into more abstract analytical categories (third order constructs). The constructs were managed with NVivo qualitative analysis software (Lumivero, Inc), enabling comparison of codes across all of the included studies. All authors met regularly to discuss codes and refine themes. Measures for transferability, credibility, confirmability, and dependability were integrated throughout the study to safeguard trustworthiness. Procedures included the sampling approach, maintenance of an audit trial, triangulation of investigators, bracketing by researchers through reflexive journaling and group meetings, and dense description of data.

## Results

### Search Outcome

A total of 177 studies were initially identified through database searches and other sources (eg, hand searches). After 8 duplicates were removed, 169 remained. Review of the abstracts and titles of the 169 studies resulted in exclusion of an additional 72. Ninety-seven full-text articles were then assessed for eligibility, and 25 met the inclusion criteria (Figure 1). Reference lists of the 25 studies were searched for relevant articles, but no additional studies were found.

### Study Characteristics

The characteristics of the 25 included studies are summarized in Table 1. Ages of the 606 total participants ranged from 18 to 89 years. Overall, cancer accounted for the most common condition related to cognitive dysfunction (10 studies).^14-23^ Cognitive dysfunction related to breast cancer was most common, in 5 studies,^15-18,20^ followed by cardiovascular disease^24-26^ and depression,^27-29^ in 3 each. Finally, experiences in post-acute sequalae of SARS-CoV-2 (PASC) or “long COVID” were explored in 2 studies.^30,31^

### Methodological Quality

All publications were reviewed by 3 authors. Some studies lacked detail in reporting methodology, and so it was challenging to compare the various methods critically. None of the studies were excluded on the basis of quality, for 3 reasons: (1) the methodological quality of each was good enough to merit inclusion; (2) the goal of this review is to provide a useful interpretation of the literature and not to locate “gold-standard” articles; and (3) although CASP has a theoretical basis in Noblit and Hare’s^45^ methodology, their original discussion refers to exclusion of studies only because they are not relevant to the research question or they are not qualitative. The results of the quality appraisal can be seen in Supplemental Material B.

### Key Themes

**Figure 2.**
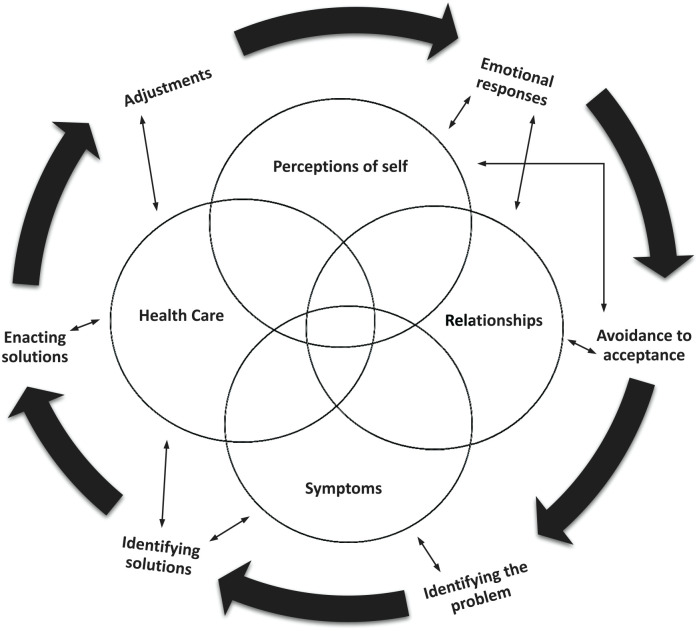
Process of Adapting (ProAdapt) model.

Across the synthesized studies, patients with subjective cognitive dysfunction were working on the process of adapting to new changes. This overarching theme encompassed 4 interrelated subthemes or areas of adaptation—specific symptoms of cognitive dysfunction, health care, perceptions of self, and relationships, which form a novel conceptual explanatory model called the ProAdapt Framework (see Figure 2 and Table 2). This model describes the central experiences of decreased cognitive function in the context of chronic illness and the ways in which participants adapt to such changes.

**Table 2.** Examples of Analytic Categories and Descriptive Themes.

Broad groups of analytic categories	Analytic categories	Examples of descriptive themes based on code groups
Process of adapting	Adapting to change Difficulties and solutions to coping Emotional responses to cognitive changes	Adapting takes energy Locating and balancing energy Acceptance of new situation Difficulty accepting new limitations Planning for self-care Working harder to perform at the same level
Symptoms	Symptoms related to cognitive change Problems with executive function, language, and attention Trajectory of symptoms	Fatigue and apathy Inability to organize thoughts Memory issues are frustrating Unprepared for changes Cognitive changes worsen over time Cognitive changes persist after physical symptoms improve
Perceptions of self	Impacts of hidden disabilities Self-conscious about perceptions from others Changes to ability affect self-esteem	Stigma Unspoken demands on self Lack of cognitive confidence Inability to live up to expectations Potential is limited Ruminating on impairment
Relationships	Work Family Social activities	Relationships with colleagues have new difficulties Fear of losing job Changes result in lower work capacity Family role changes Tension between prioritizing family and self Discrepancy between perceived ability and family’s perception Dissatisfied with changes in ability to socialize
Health care	Cognitive testing Lack of help from HCPs Condition (eg, COPD) specific information related to cognitive change	Cognitive testing is a part of treatment and leads to proper care Health care providers dismissed concerns Want to hear from HCPs with expertise Fear of cognitive changes affecting medical care Desire for clear information and care plans

Abbreviation: COPD: chronic obstructive pulmonary disease; HCP: health care provider.

#### Process of adapting

In adapting to new changes, participants expressed that their everyday lives were more challenging and that performing routine tasks was difficult because their ability to make decisions was affected. As they recognized these changes and their own limitations, they negotiated with themselves about activities and allocated their energy throughout the day by making priorities:

If . . . it’s dusty then there will be a negotiation. It costs a lot of energy to dust then I negotiate with myself then someone else will do it, usually I would have dusted and cooked food at the same time but now I make a priority because then I think it’s better that I have energy for the children when they get home.^24(p230)^

In addition, new changes resulted in patients’ needing to develop new solutions. They planned for self-management and worked harder to function at their prior levels, because their cognitive dysfunction affected their self-management skills and made simple tasks difficult.^4,5,14,18,32^ However, their strategies for self-management were not always effective:

I think I have problems remembering to take my medications, but it does not benefit me to mark on calendars or anything like that. I even have a pill that I have to take an hour after I eat and I’ll remember it past the hour and it’s too late.^4(p491)^

Denial became a coping strategy and was present in various ways. For some, this meant not dealing with newly developing cognitive changes until they could finally not be ignored. Although patients were eventually forced to recognize their cognitive changes and find solutions, many found it difficult to accept their new limitations.^19,30,31^ They also sidestepped everyday activities, choosing to “ignore their difficulties because they found it hard to fully accept their limitations”^24(p230)^ and to “avoid confrontation with a difficulty.”^33(p2360)^ They also stated a desire to regain their prior capabilities, wanting to “be like I used to be, normal.”^25(p1229)^

#### Symptoms

Among the specific symptoms of subjective cognitive dysfunction that patients reported, problems with memory, executive function, language, communication, and attention stood out. Memory issues affected every situation, as one participant explained: “Well, I can remember when I used to drive for [a bus company] one time I couldn’t even remember where I was at. I forgot. Wrong turns and stuff like that.”^4(p490)^ Other participants complained about “word-finding difficulties”^33(p2354)^ and said that it was hard to remember people’s names, appointments, and medications.^34^ Patients also had difficulties with focus and attention. They could not keep their minds focused for long periods: “I try to focus on something for literally 10 minutes, I just find myself 5 minutes later, going ‘well I was reading that thing and then what happened.’”^28(p325)^ Such difficulties with memory and attention were found to affect a person’s communication with other individuals, because the person could not remember prior conversations or other people’s names.^14,31,32^

Other symptoms that were related to cognitive change included fatigue and apathy, with the fatigue making patients’ cognitive function worse: “When I’m tired it gets worse. The fatigue . . . just yesterday I was at a friend’s house and . . . I have to take a power nap, in the middle of the day . . . and I didn’t get that.”^18(pE37)^ As such, fatigue greatly affected individuals’ daily activities and forced many to reduce their responsibilities and set new boundaries. For example, fatigue was one of the reasons for many patients’ early retirement from work.^32^ In another example, the co-occurrence of fatigue and apathy was clear: “I think a lack of energy but a lack of interest as well [is a problem].”^33(p2354)^

Cognitive symptoms that resulted from chronic conditions were unexpected by patients, because health care providers had not informed them about the potential for cognitive changes, leaving them unprepared.^14^ Some even thought that this process was “just a normal part of getting old.”^5(p4)^ The trajectory of cognitive changes varied with patients, but most perceived that their cognitive dysfunction had worsened over time and persisted: “I think it’s gotten worse over the years,”^16(p724)^ and “It’s becoming more and more.”^28(p466)^

#### Perceptions of self

Central to explanations of how changes in cognitive function affected individuals’ lives were changes in self-perception. Feelings of loss, changes in usefulness, sense of worth, and symptoms all limited perceived future potential. Individuals’ cognitive symptoms demanded new expectations about their life course. This led to increased stress, due in large part to unspoken demands and expectations in family and everyday life. Participants ruminated on such changes in how they viewed themselves, and frequently their negative thoughts interfered further with their already impaired abilities to concentrate. “It’s probably like a compounded thing but mostly you’re just sad and low energy . . . just adds to the hopelessness and self-hatred or whatever.”^27(p326)^

#### Relationships

Participants in several studies reflected on changes to relationships due to decreased cognitive functioning. Affected relationships included those in the workplace, with family at home, with friends in social situations, and with others with chronic illnesses. Participants were worried about burdens they now placed on work colleagues as well as family^14,21,25,27,33^: “I take time off work which isn’t good, I just have said I’m having a mental health day and I’ve told them I’ve been to the doctor”^27(p326)^; “I’m not this mother anymore who fixes everything they used to.”^24(p230)^ Others were concerned about the impact that “hidden” symptoms had on relationships: “Many [symptoms] unfortunately cannot be seen and then they do not exist . . . if you are doing something with others . . . they do not understand if you get tired . . . if it gets tough.”^25(p6)^

#### Health care

Interactions with health care providers were prominently featured across most studies, with 4 main areas of focus: (1) discussions of testing for cognitive dysfunction, (2) issues with health care providers’ responses to cognitive symptoms, (3) desire for health care providers’ expertise, and (4) concern about how health care providers might view cognitive changes. Cognitive testing was thought to be a part of treatment that would lead to proper care.^32,34^ Screening was thought be part of “normal” treatment if offered by health care providers.^34^ However, health care providers’ dismissal of cognitive complaints was common.^4,5,14,15,28,30,31^ Thus, one participant described how he had searched the internet on his own, yet when he asked his provider about cognitive changes that he had been experiencing, his concerns were “pushed to the side.”^4(p4)^ Some patients rationalized this: “I guess it isn’t a high priority, it’s low on the list. When you have a breathing problem, it’s like they say, if you can’t breathe nothing else matters.”^34(p1235)^ Conversely, information overload was unhelpful but common. Patients talked of being overwhelmed by handouts, notebooks, and pamphlets about side effects of medical treatments or complications of chronic conditions—much of which went unread. And what was read did not address cognitive changes. “There was nine million things in there about support and you know family and blah, blah, blah. It’s just like this is over the top. Was someone really advised of something when you’ve given them this boxcar of paper and one line in the middle?”^14(pp10,11)^

Despite these challenges, patients wanted to hear from health care providers with expertise—in part to hear about potential risks and options for treatment, but also for reassurance that they were not “crazy” or alone in their struggles.^5,14,15,18,21,24,35^ Patients wanted to talk about cognitive changes with their health care providers so that they could understand what was happening and get “the right kind of help.”^14(p10)^ They emphasized that a consistent relationship with a provider who was familiar would be helpful. “I been with him [physician] now . . . at least 10 or 15 years . . . we sit there and we talk . . . he mostly takes time . . . tries to explain medical situations to you . . . and what you need to do.”^35(p205)^ Without this long-standing relationship, patients were afraid that no one would take their cognitive changes seriously until they became severe. “It’s not until you are in a very severe state with your cognition that you actually get attention and by then it’s too late.”^32(p5)^

## Discussion

To the best of our knowledge, this is the first synthesis of qualitative studies examining subjective cognitive function in adults with nondementia-related chronic illness. The conceptual explanatory model derived from our analysis of the data in this study consists of 4 areas or patterns of interrelationships. As Hoyt and Stanton^36^ have noted, the dimensions of adjustment in chronic illness are interrelated, multidimensional, and apparent across disease processes. We found those dimensions within the predominant theme of “the process of adapting,” which contained a sense of loss and “making the best of situations” in coping with new realities in relationships, health care, and self-perceptions (Supplemental Material B). As depicted in the model, the adaptations that participants described changed over time and did not follow a linear path. Instead, participants were continually identifying problems and solutions and then making adjustments.

Across illnesses, the symptoms of cognitive dysfunction were described by the participants in the reviewed studies as both physical (“my brain hurts”) and mental (“my thinking is foggy”) and were defined by them as indicative of a change in health status. Symptom theory has defined symptoms similarly, as subjective experiences based on individuals’ perceptions^37^; these subjective experiences include detection, interpretation, and responses to alterations in health status. Henly et al^38^ defined the experience of symptoms as a “flow”—a process of constant evaluation and reevaluation of timing, force, quality, and distress. The cyclical characteristics of the themes described in the present meta-synthesis’ model reflect that flow. Adding a layer of complexity, the interpretation of symptoms is subject to cognitive resources,^39^ which for the participants in this review were already limited. Chronic illnesses are often studied from a self-management perspective, but what if they were studied from an integrated symptom/self-management approach instead? Such an approach would allow a more thorough, holistic understanding of the impact of symptoms such as cognitive dysfunction on self-management and the influence of self-management on symptoms.

Some participants were not able to recapture some of their perceived losses from cognitive dysfunction and reported that they were still struggling with changes in roles; their self-perceptions changed dramatically. In the literature on experiences of chronic illness, positive experiences in which individuals discover meaning or strength from their struggles are frequently reported.^40^ Among participants in the present study, however, distress was more predominant and positive depictions were limited. This is likely a result of a lack of formal diagnosis, which keeps patients from “making sense” of their difficulties,^43^ and it is similar to findings for other conditions that are rare or difficult to diagnose, such as endometriosis^41^ and lupus.^49^

As depicted in the interrelationships in the model, the participants in this meta-synthesis continually described an overlap between the effect of symptoms and self-management of not only illness, but relationships with family and work. In 2 studies, for example, participants with diabetes mentioned the effect of perceived cognitive dysfunction on diabetes self-management and said that their management of diabetes affected their cognitive symptoms (eg, eating high-carb foods led to “brain fog”).^4,5^ In addition to the practical struggles of chronic illness management, the participants’ self-perceptions were altered and included altered expectations with regard to how they might fit into their prior world. In other studies, having a chronic illness has become a significant identity.^50-52^ Perceptions of goals and responsibilities, as well as perceived causes of chronic illness, influenced participants’ sense of self and adaptation. The emotional impact of this is a concern. Participants expressed mostly negative thoughts about their changed self-perceptions and their potential social isolation; not only chronic illness alone, but chronic illness affected by the stigma of cognitive dysfunction can be a source of stress and depression.^53-55^ With regard to cognitive dysfunction, the role of family members in providing support has received attention in the literature, but this attention has focused mostly on those acting as “caregivers” of those with dementia or Alzheimer’s disease.^56,57^ None of the participants in the reviewed studies referred to family members, friends, or other support as caregivers. In fact, one participant mentioned that “caregiver” was a term used to describe someone who took care of a person with dementia.^5^ Some participants were hiding symptoms to avoid such a situation and were actively working to avoid “becoming a burden.”^14,21,25,26,33^

These results advance the theoretical understanding of subjective cognitive dysfunction in the context of chronic illness and provide insights into how health care providers can support patients as they attempt to deal with these issues. Due to the nature of chronic illnesses and the need for frequent clinic appointments, clinicians who work with patients who have chronic illnesses should use such opportunities to speak to patients about cognitive dysfunction and how they may be affecting the trajectory of the patient’s chronic condition, overall health, and quality of life. The model proposed here suggests a need for health care providers to frequently reassess cognitive symptoms in chronic illness by recognizing a more dynamic trajectory: what works today may not work tomorrow, or what worked last year may work again today. To recognize and support patients’ experiences of chronic illness, one must meet people in the moments along such trajectories. Additional clinical gains from early recognition of cognitive dysfunction include motivation to plan for the future; obtain support; assess for possible safety issues, such as the need for assistance with medications; and participate in research to find new therapies for cognitive dysfunction.^58^ There are also cost benefits to early detection, with recent evidence of lower overall health care costs in the year following diagnosis of dementia with appropriate pharmacological treatment.^59^

Meta-synthesis involves interpretation, and other researchers might come to different conclusions by using a different framework. However, the interpretive aspect of meta-synthesis allows researchers to consider theories that may or may not have been presented in the original reviewed publications. In applying the CASP criteria across the reviewed studies, there was some variation: some studies met the criteria minimally, whereas other studies did so more robustly. However, operationalizing more specific criteria would diminish the flexibility that allows the inclusion of different assumptions of various methodological approaches. An additional limitation to the present meta-synthesis is that we analyzed, for practical reasons, only articles in English. Studies from non–English-speaking or non-Western countries should also be analyzed, because culture may influence the experience of cognitive dysfunction. Older studies (10 years or older) were included in the analysis. This may be considered a limitation as treatments for cognitive problems in chronic illness may have changed over time. However, we made a purposeful effort to include perspectives of participants from underrepresented groups and 5 of the older studies examined perspectives of women and Black participants. Therefore, the decision was made to include all applicable studies. All of the synthesized studies used 1-time data collection methods, and so longitudinal qualitative data methods used in other areas of chronic illness would also be beneficial. After all, the experience of cognitive dysfunction changes over time. In addition, samples varied across studies. The nature of each chronic illness, length of time since diagnosis, and self-management expectations may have an effect on participants’ responses. Research that focuses on one diagnosis with participants in similar stages may provide more detailed information about individual experiences at particular points in the disease process.

In this meta-synthesis, we have reviewed the literature on the subjective experience of cognitive dysfunction in the context of chronic illness and identified one main theme, the process of adapting, and 4 subthemes: (1) perceptions of self; (2) symptoms; (3) relationships; and (4) health care. The somewhat unexpected nature of cognitive dysfunction was problematic for participants, families, and, in the participants’ views, health care providers. The participants in these studies used a variety of coping strategies; but these strategies appeared to have limited benefits, and they will require further investigation to inform helpful self-management interventions. Given the findings of this study, the potential effects of chronic illnesses on cognitive function need to be better communicated with these patients to better support them. Both researchers and health care providers need to examine what experiences patients are describing as positive. Health care providers may face challenges in working with these patients, whether because cognitive dysfunction may not be their area of expertise (eg, endocrinologists working with people with diabetes) or because they are unfamiliar with current therapies or guidelines for screening for cognitive dysfunction in chronic illness.^60-62^ Thus, further education and training of health professionals not only to manage but also to validate and acknowledge cognitive dysfunction are needed. At the same time, more evidence is needed to inform effective interventions to help reduce cognitive dysfunction related to cognitive illness.

## Supplemental Material

sj-docx-1-wjn-10.1177_01939459241272039 – Supplemental material for Subjective Cognitive Dysfunction in Chronic Illness: A Systematic Review and Meta-Synthesis Supplemental material, sj-docx-1-wjn-10.1177_01939459241272039 for Subjective Cognitive Dysfunction in Chronic Illness: A Systematic Review and Meta-Synthesis by Heather Cuevas, Elizabeth Heitkemper and Jeeyeon Kim in Western Journal of Nursing Research

sj-docx-2-wjn-10.1177_01939459241272039 – Supplemental material for Subjective Cognitive Dysfunction in Chronic Illness: A Systematic Review and Meta-Synthesis Supplemental material, sj-docx-2-wjn-10.1177_01939459241272039 for Subjective Cognitive Dysfunction in Chronic Illness: A Systematic Review and Meta-Synthesis by Heather Cuevas, Elizabeth Heitkemper and Jeeyeon Kim in Western Journal of Nursing Research
