# Supplementary material for: Subjective Cognitive Dysfunction in Chronic Illness: A Systematic Review and Meta-Synthesis
Source: West J Nurs Res. 2024 Aug 19;46(9):708–24. doi: 10.1177/01939459241272039 (PMC11380369; doi:10.1177/01939459241272039)
Supplement: sj-docx-1-wjn-10.1177_01939459241272039 – Supplemental material for Subjective Cognitive Dysfunction in Chronic Illness: A Systematic Review and Meta-Synthesis [file sj-docx-1-wjn-10.1177_01939459241272039.docx]

*Supplemental Material B*: CASP - Quality Appraisal of Studies

| Author,  Year | Valid results | Appropriate method | Appropriate design | Appropriate recruitment | Appropriate data collection | Researcher & Participant relationship addressed | Considered ethical issues | Rigorous analysis | Clear statement of findings | Value of research |
| --- | --- | --- | --- | --- | --- | --- | --- | --- | --- | --- |
| Alenljung,  2019 |  |  |  |  |  |  |  |  |  |  |
| Bolt,  2020 |  |  |  |  |  |  |  |  |  |  |
| Callan, 2022 |  |  |  |  |  |  |  |  |  |  |
| Chasco, 2022 |  |  |  |  |  |  |  |  |  |  |
| Crouch, 2017 |  |  |  |  |  |  |  |  |  |  |
| Crowe, 2020 |  |  |  |  |  |  |  |  |  |  |
| Cuevas, 2017 |  |  |  |  |  |  |  |  |  |  |
| Cuevas, 2018 |  |  |  |  |  |  |  |  |  |  |
| Disler, 2020 |  |  |  |  |  |  |  |  |  |  |
| Heli, 2015 |  |  |  |  |  |  |  |  |  |  |
| Klymko, 2011 |  |  |  |  |  |  |  |  |  |  |
| Kudlicka, 2018 |  |  |  |  |  |  |  |  |  |  |
| Lelorain,  2012 |  |  |  |  |  |  |  |  |  |  |
| McAuliffe, 2019 |  |  |  |  |  |  |  |  |  |  |
| Miebach, 2019 |  |  |  |  |  |  |  |  |  |  |
| Meibach,  2018 |  |  |  |  |  |  |  |  |  |  |
| Munir,  2010 |  |  |  |  |  |  |  |  |  |  |
| Munir, 2011 |  |  |  |  |  |  |  |  |  |  |
| Myers,  2012 |  |  |  |  |  |  |  |  |  |  |
| Pappadis, 2019 |  |  |  |  |  |  |  |  |  |  |
| Potrata, 2010 |  |  |  |  |  |  |  |  |  |  |
| Rust, 2013 |  |  |  |  |  |  |  |  |  |  |
| Sawyer, 2016 |  |  |  |  |  |  |  |  |  |  |
| Wu, 2013 |  |  |  |  |  |  |  |  |  |  |
| Zeng, 2017 |  |  |  |  |  |  |  |  |  |  |

Key

|  | Yes |
| --- | --- |
|  | No |
