# Supplementary material for: Subjective Cognitive Dysfunction in Chronic Illness: A Systematic Review and Meta-Synthesis
Source: West J Nurs Res. 2024 Aug 19;46(9):708–24. doi: 10.1177/01939459241272039 (PMC11380369; doi:10.1177/01939459241272039)
Supplement: sj-docx-2-wjn-10.1177_01939459241272039 – Supplemental material for Subjective Cognitive Dysfunction in Chronic Illness: A Systematic Review and Meta-Synthesis [file sj-docx-2-wjn-10.1177_01939459241272039.docx]

**Supplementary Material A**

| **Review question:** | - What are the experiences of subjective cognitive dysfunction in people with non-neurologic chronic disease? |
| --- | --- |
| **Population:** | - Participants with non-neurologic chronic illnesses such as diabetes, cardiovascular disease and arthritis. |
| **Exposure:** | - N/A |
| **Comparators:** | - N/A |
| **Outcomes:** | - Must report primary study data or outcome data related to subjective cognitive function. |
| **Inclusion criteria:** | - Primary study or secondary analysis/meta-analysis of primary study data [data-driven] - Peer-reviewed - English-language only - Published through June 2023 - Population of interest includes individuals with non-neurologic chronic disease. |
| **Exclusion criteria:** | - Non data-driven study - Not peer-reviewed - Not written in English - Study does not focus on either 1) subjective cognitive function, or 2) focuses on quantitative results. - Study includes only participants with Alzheimer’s disease, stroke, fibromyalgia, traumatic brain injury or other neurologic conditions. |
| **Study selection:** | - Overall, 112 studies were identified through database searching and through other sources. - After duplicates were removed, 106 studies remained. - 66 were excluded by title and abstracting screening. - 40 studies were assessed for eligibility via full-text screening resulting in a total of 25 studies for analysis. |
| **Risk of bias assessment:** | - The quality of included studies was assessed with the Critical Appraisals Skills Programme (CASP, 2020). - Study quality was independently assessed by three reviewers using this methodological quality appraisal checklist. |

**Search Strategy**

**Table 1. Medline search strategy**

| 1 | Chronic illness [Title/Abstract] OR chronic disease [Title/Abstract] OR non-dementia [Title/Abstract] |
| --- | --- |
| 2 | Subjective cognitive*[Title/Abstract] OR self-reported*[Title/Abstract] OR cognitive dysfunction [Title/Abstract] OR cognition*[Title/Abstract] OR subjective cognitive dysfunction [Title/Abstract] OR cognitive function* [Title/Abstract] OR cognitive impairment* [Title/Abstract] OR interview [Title/Abstract] OR qualitative [Title/Abstract] OR mixed methods [Title/Abstract] |
| Filters | Publication date through January 2022; Humans; English |

**Table 2. CINAHL search strategy**

| 1 | Chronic illness OR chronic disease OR non-dementia |
| --- | --- |
| 2 | Subjective cognitive* OR self-reported cognitive* OR cognitive dysfunction OR cognition* OR subjective cognitive dysfunction OR cognitive impairment* OR cognitive impairment* OR cognitive function* OR qualitative interview* OR mixed methods |
| Filters | Peer-reviewed, humans, English |

**Table 3. PubMed search strategy**

| 1 | Chronic illness [Title/Abstract] OR chronic disease [Title/Abstract] OR non-dementia [Title/Abstract] |
| --- | --- |
| 2 | Subjective cognitive*[Title/Abstract] OR self-reported*[Title/Abstract] OR cognitive dysfunction* [Title/Abstract] OR cognition*[Title/Abstract] OR subjective cognitive dysfunction [Title/Abstract] OR cognitive function* [Title/Abstract] OR interview [Title/Abstract] OR qualitative [Title/Abstract] OR mixed methods [Title/Abstract] |
| Filters | Publication date through January 2022; Humans; English |

**Table 4. EMBASE search strategy**

| 1 | Chronic illness [Title/Abstract] OR chronic disease [Title/Abstract] OR non-dementia [Title/Abstract] |
| --- | --- |
| 2 | Subjective cognitive*[Title/Abstract] OR self-reported*[Title/Abstract] OR cognitive dysfunction [Title/Abstract] OR cognition*[Title/Abstract] OR cognitive impairment* [Title/Abstract] OR cognitive function* [Title/Abstract] OR subjective cognitive dysfunction [Title/Abstract] OR interview [Title/Abstract] OR qualitative [Title/Abstract] OR mixed methods [Title/Abstract] |
| Filters | Publication date through January 2022; Humans; English |

**Data Extraction**

| 1 | Title |
| --- | --- |
| 2 | First author |
| 3 | Funding source |
| 4 | Possible conflicts of interest listed? (yes/no, if yes please list) |
| 5 | Addresses recruitment strategy? (yes/no) |
| 6 | Addresses retention strategy? (yes/no) |
| 7 | Addresses participant views on research? (yes/no) |
| 8 | Is the study guided by a theory or conceptual framework? (yes/no) |
| ***Demographics*** | |
| 9 | Does study focus on a specific population or subgroup? (yes/no, if yes please list) |
| 10 | Does study focus on participants living in a specific setting? – community, assisted living facility, skilled nursing facility, acute care |
| 11 | Inclusion criteria |
| 12 | Exclusion criteria |
| 13 | Total number of participants |
| 14 | Average age |
| 15 | Male (n, %) |
| 16 | Female (n, %) |
| 17 | White (n, %) |
| 18 | Racial/ethnic group membership |
| 19 | Aim of study |
| 20 | Design of study |
| 21 | Did the study use any questionnaires and/or tools? If so, note name here. |
| ***Subjective cognitive function*** | |
| 22 | Assessment schedule |
| 23 | Subjective cognitive function results |
| 24 | Study findings |
| 25 | Study aim |
| 26 | Other key findings |
| 27 | Applicability |
| 28 | Have important populations been excluded from the study? |
| 29 | Do study findings directly address the review question? |
| ***Conclusion*** | |
| 30 | Key conclusion |
| 31 | Correspondence required for further study information? |
| 32 | Contact |
| 33 | Correspondence received |
| 34 | Notes |
| ***Other*** | |
| 35 | Study should be excluded as it does not meet eligibility criteria – put in check list of ineligibility |
